# Community group membership and multidimensional subjective well-being in older age

**DOI:** 10.1136/jech-2017-210260

**Published:** 2018-02-09

**Authors:** Daisy Fancourt, Andrew Steptoe

**Affiliations:** Department of Behavioural Science and Health, University College London, London, UK

**Keywords:** quality of life, social activities, psychosocial factors

## Abstract

**Background:**

It has been highlighted as a public health priority to identify ways of supporting well-being in older age to allow people to lead healthy and integrated lifestyles. This study explored whether membership in eight different sorts of community groups was associated with enhanced experienced, evaluative and eudemonic well-being among older adults.

**Methods:**

We analysed data from 2548 adults aged 55+ drawn from the English Longitudinal Study of Aging. We used multivariate logistic and linear regression models to compare change in well-being between baseline and follow-up 10 years later in relation to membership of different community groups while controlling for potential confounding variables.

**Results:**

Membership in two types of community groups was associated with enhanced well-being: attending education, arts or music classes was longitudinally associated with lower negative affect (OR 0.73, CI 0.57 to 0.92) and life satisfaction (β 0.55, CI 0.02 to 1.08) while church or religious group membership was longitudinally associated with lower negative affect (OR 0.79, CI 0.65 to 0.98) and higher positive affect (OR 1.54, CI 1.25 to 1.90). There was no evidence of reverse causality through cross-lagged analyses. However, just 17.4% and 24.6% of older adults were found to engage in these two types of community groups, respectively, and several demographic factors were identified as barriers to participation.

**Conclusions:**

Overall, this study suggests that education, arts or music classes and church or religious groups may support well-being in older age. Programmes to encourage engagement could be designed for older adults who may not normally access these community resources.

## Introduction

It has been highlighted as a public health priority to identify ways of supporting well-being in older age to allow people to lead healthy and integrated lifestyles.^1^ Subjective well-being has been identified as an important area of focus not just for the impact it has on each individual’s quality of life but also because it has been shown to have a bidirectional relationship with physical health. Older people with illnesses such as coronary heart disease, arthritis and chronic lung disease show impaired hedonic and eudemonic well-being, but also those with poorer eudemonic well-being have higher concentrations of inflammatory markers, poorer lung function and shorter survival on average.^2 3^

Well-being is broadly recognised as a multidimensional structure,^4^ frequently split into hedonic (relating to experiences of pleasure) and eudemonic well-being (relating to human flourishing).^4^ Hedonic well-being is further categorised into evaluative well-being (relating to life satisfaction) and experienced or affective well-being (subcategorised into positive affect and negative affect). For eudemonic well-being, subcategories cluster around concepts of control, mastery and autonomy, and personal growth, competence and self-realisation.^5^ In older age, studies from high-income English-speaking countries have shown that evaluative well-being increases slightly in older age while stress and worry fall.^3^ However, despite these positive broad trajectories, other analyses of England specifically have suggested that there are in fact within-subject declines in well-being as people age.^6^

Consequently, there has been particular interest in identifying activities that could support the well-being of older adults. One strand of work has focused on the well-being benefits of community group membership.^7^ Groups have been identified as sources of personal security, social companionship, emotional bonding, intellectual stimulation, collaborative learning, collective goal attainment, self-esteem and sense of worth.^8^ Research into group membership and well-being in older age has focused most on the well-being impact of identity loss through leaving groups such as retirement from work.^9–12^ However, there is also increasing interest on potential positive effects for well-being among older adults of engagement in community-run clubs, societies and groups,^13^ including church attendance,^14^ charitable volunteering,^15^ environmental volunteering,^16^ social clubs,^17^ music, singing and drama groups,^18^ and sports clubs and gym membership.^19^

However, there are several limitations to existing research. First, a number of existing studies have been cross-sectional rather than longitudinal, which means causality is very unclear: people with higher levels of well-being may be more likely to choose to engage in groups. Second, members of one group are likely to be members of other groups too, and membership of multiple groups appears to be protective against the development of depression and for the maintenance of well-being.^20^ Consequently, as many previous studies have not simultaneously tested for the effects of multiple types of groups, it is questionable as to whether they have genuinely been measuring the effects of the specific groups they describe or just more general ‘group engagement’. Third, many previous studies have looked at broad associations between group membership and ‘well-being’ as a unidimensional construct, but a comparative assessment of different types of club and society membership and multidimensional well-being remains to be undertaken. Finally, we still have very little detail about which community group’s older adults engage with and what demographic factors affect their likelihood of engaging. Consequently, this study sought (i) to profile the engagement of older adults in eight different types of community groups and (ii) to explore longitudinal associations between baseline and follow-up 10 years later between membership in these community groups and five different subcategorisations of well-being.

## Methods

### Participants

Participants were drawn from the English Longitudinal Study of Aging: an ongoing nationally representative cohort study of community-dwelling adults aged ≥50 that started in 2002. In this analysis, participants were core respondents drawn from wave 2 (2004/2005) and followed up 10 years later at wave 7 (2014/2015). We specifically analysed data from participants aged ≥55 who provided data on all variables included in our models, excluding any who were registered blind. This provided a total sample of 2548 participants.

### Measures

Well-being was assessed using five different scales, as recommended by a specific factor analysis of measures included within the English Longitudinal Study of Aging (ELSA).^21^ For experienced well-being, negative affect was measured using the short Centre for Epidemiologic Studies Depression Scale (CES-D): an eight-item scale measuring mood and somatic aspects of depression.^22^ As we were focusing on negative affect rather than clinical depression, we dichotomised scores into no symptoms (45% of respondents) versus present symptoms (55% of respondents). Positive affect was measured using the pleasure subcomponent of the scale CASP-15.^23^ As with negative affect, this variable was dichotomised into maximum (49% of participants) versus submaximum positive affect (51% of participants). Evaluative well-being was measured using the Satisfaction with Life Scale^24^; a five-item scale measuring an idiosyncratic judgement of an individual’s life. Hedonic well-being was measured using the two remaining subcomponents of CASP-15: control/autonomy and self-realisation.

Participation in different social clubs was measured through eight binary response questions asking whether participants were a member of (i) political party, trade union or environmental groups; (ii) tenant groups, resident groups, neighbourhood watch groups; (iii) church or other religious groups; (iv) charitable associations; (v) education, arts or music groups or evening classes; (vi) social clubs; (vii) sports clubs, gyms, exercise classes; and (viii) any other organisations, clubs or societies. While asking individuals about *frequency* of engagement in activities can be somewhat subjective, asking about active *membership* is a more objective measure of engagement, so it was felt to be a suitable independent variable for this analysis.

Previous research has suggested that engagement in different types of clubs and societies is strongly associated with a range of demographic variables. So analyses were adjusted for all demographic variables identified as being potentially associated both with club/society membership and well-being (and therefore constituting potential confounders). This included sex, age, marital status (married/cohabiting vs never married/divorced/separated/widowed), ethnicity (white or other), educational attainment (completed education prior to the age of 16, education up to age 16, education up to age 18, and higher education past the age of 18), employment status (not working vs working) and wealth (split into quintiles, referring to total net non-pension assets; identified as a robust indicator of socioeconomic circumstances in ELSA analyses).

In addition, engagement in clubs and societies is also likely to be affected by health-related variables, which can also affect well-being (constituting further confounders). So our analyses further adjusted for eyesight and hearing (both scored as excellent/very good/good vs fair/poor), presence of a long-standing illness (including cancer, chronic obstructive pulmonary disease, arthritis, diabetes, angina or having experienced a stroke in the past two years) and chronic pain (moderate/severe vs mild/none).

### Statistics

To explore our first research question and profile the engagement of older adults in eight different types of community groups, we used χ^2^ tests to explore whether there were demographic differences between people who were or were not member of each of the eight types of groups we focused on.

To explore our second research question and ascertain whether there were longitudinal associations between membership of community groups and well-being, we then used multivariate regression models (linear and logistic depending on whether each well-being outcome was continuous or binary). In order to minimise the number of tests carried out and control for membership in other differing types of groups, all eight community groups were tested in the same regression analysis. Our analyses controlled for all identified confounding variables: model 1 adjusted just for baseline well-being along with identified demographic confounders outlined above, and model 2 additionally adjusted for identified health-related confounders outlined above.

To test the robustness of our models, we carried out a series of sensitivity analyses. First, we explored whether the number of times a person engages with their club or society affects the relationship with well-being by additionally adding a count of attendance at all clubs combined over the past 12 months into our regression analyses. Second, we explored whether it was specifically the effects of *formal* group membership or broader *informal* activity associated with well-being by additionally adding two further variables into the analyses: a binary variable as to whether participants had a further hobby or pastime and a continuous variable measuring the frequency of social contact with non-cohabiting relatives. We selected relatives rather than friends as social contact with friends could be affected by group membership so it is likely to lie on the causal pathway between group membership and well-being. Therefore, non-cohabiting relatives provided an appropriate proxy not likely to lie on the causal pathway. Third, we explored whether mobility issues might affect results by excluding participants who reported problems with walking. Fourth, in order to account for attrition across the 10 years, we ran additional sensitivity analyses applying inverse probability weighting using cross-sectional weights. This also reweighted the data to ensure it remained nationally representative. Finally, we wanted to ascertain whether results varied as people aged, so we split participants by age (55-64 and 65+) and reran analyses. Results are provided in online supplementary tables S1–S5.

10.1136/jech-2017-210260.supp1 Supplementary file 1

To test the possibility of reverse causality in our analyses, even when taking into consideration baseline well-being and covariates, we reran analyses for all significant findings, reversing the independent and dependent variables. These cross-lagged analyses allowed us to explore whether baseline well-being predicted group membership 10 years later, even when additionally controlling for baseline group membership, and test whether our results showed evidence of being bidirectional.

## Results

### Engagement

Table 1 shows the percentage of each demographic (eg, men, women, those working, those not working etc) who are members of each type of group. The most common community groups to be a member of are sports clubs, gym or exercise classes (with 26% of participants overall reporting being a member), closely followed by church or religious groups (24.6%), tenant groups, resident groups or neighbourhood watch groups (23.4%), charitable associations (21.9%), social clubs (20%) or ‘other’ clubs (19.6%). Education, arts, music or evening classes (17.4%) and political party, trade union or environmental groups (15.7%) are the least common community groups to be involved in. Results suggest that there is a gender split between types of groups that older adults engaged in, with men more likely than women to engage in social clubs and tenant groups, resident groups and neighbourhood watch groups, but women more likely to engage in education, arts or music groups or church or religious groups. There is also evidence of a difference in age, with adults aged 65+ more likely to engage in all groups except political party, trade union or environmental groups, or sports clubs, gyms or exercise classes. There is also evidence of a social gradient across all forms of group membership, with those of higher socioeconomic status more likely to engage in all group except social clubs, for which the gradient was reversed.

**Table 1:** Engagement in clubs and societies by demographic characteristics

	Education, arts, music	Church or religious	Sports or exercise	Charitable	Social clubs	Political, union, environment	Resident or neighbourhood	Other
All (%)	17.40	24.60	26.00	21.90	20.00	15.70	23.40	19.60
Sex								
Men (%)	13.20	18.90	25.90	20.40	23.4	19.00	25.00	33.80
Women (%)	20.80	29.30	26.10	23.10	17.20	13.10	22.00	26.80
	χ^2^=25.4***	χ^2^=37.2***	χ^2^=0.01	χ^2^=2.80	χ^2^=14.9***	χ^2^=16.4***	χ^2^=3.0	χ^2^=15.0***
Age (years)								
55-64 (%, n=1504)	15.82	20.2	27.0	20.5	18.6	17.3	20.9	27.9
65+ (%, n=1044)	19.64	31.0	24.6	23.9	22.0	13.5	26.8	33.0
	χ^2^=6.2*	χ^2^=39.4***	χ^2^=1.8	χ^2^=4.1*	χ^2^=4.5*	χ^2^=6.6**	χ^2^=12.6***	χ^2^=7.6**
Employment								
Not working (%, n=1492)	19.0	28.0	26.3	23.8	19.8	11.6	26.3	30.8
Working (%, n=1056)	15.1	19.8	25.6	19.1	20.3	21.6	19.2	28.7
	χ^2^=6.8**	χ^2^=22.5***	χ^2^=0.2	χ^2^=7.9**	χ^2^=0.1	χ^2^=46.6***	χ^2^=17.2***	χ^2^=1.3
Marital status								
Not coupled (%, n=618)	19.7	24.6	22.8	19.6	22.2	14.4	21.0	29.6
Married or cohabiting (%, n=1930)	16.6	24.6	27.1	22.6	19.3	16.2	24.1	30.1
	χ^2^=3.2	χ^2^<0.001	χ^2^=4.4*	χ^2^=2.5	χ^2^=2.4	χ^2^=1.1	χ^2^=2.4	χ^2^=0.04
Education								
Education: NVQ1 (%, n=718)	8.9	18.5	16.0	12.4	23.3	8.8	15.5	17.8
NVQ2 (%, n=544)	16.2	22.4	29.0	21.5	19.1	15.6	23.9	32.4
NVQ3 (%, n=837)	16.9	28.2	26.6	24.6	21.3	16.0	26.2	33.1
Degree (%, n=449)	33.4	30.3	37.2	32.3	13.6	26.5	30.1	40.5
	χ^2^ =116.8***	χ^2^=29.3***	χ^2^=69.2***	χ^2^=70.0***	χ^2^=17.4***	χ^2^=65.5***	χ^2^=40.1***	χ^2^=79.7***
Wealth								
First (%, n=496)	11.1	21.0	13.3	15.3	21.4	11.3	14.1	15.1
Second (%, n=508)	13.8	21.9	23.0	14.8	19.5	15.0	17.1	26.8
Third (%, n=498)	14.5	25.3	23.7	20.3	24.3	16.3	21.3	32.3
Fourth (%, n=514)	22.6	29.2	32.9	26.7	19.8	19.5	30.0	35.8
Fifth (%, n=532)	24.4	25.6	36.3	31.6	15.4	16.5	33.5	38.9
	χ^2^=49.3***	χ^2^=11.8*	χ^2^=87.0***	χ^2^=64.4***	χ^2^=13.4**	χ^2^=13.3**	χ^2^=78.7***	χ^2^=84.5***

*P <0.05, ** P<0.01, *** P<0.001.

### Well-being

In examining baseline associations between the five dimensions of well-being, there was a clear association between them all (see table 2). As expected, negative affect was negatively correlated with all other measures of well-being. Positive affect showed large positive correlations with life satisfaction, control autonomy and self-realisation. Life satisfaction additionally showed a large positive correlation with control autonomy and self-realisation, which also showed a large positive correlation with each other. However, as anticipated based on previous studies, these correlational analyses showed that although the five different dimensions of well-being were correlated, only 15%–45% of the variance in each dimension was explained by another dimension. As large proportions of unexplained variance remain, this confirmed that it is valuable to consider each dimension as a separate outcome variable rather than collapsing the dimensions together.

**Table 2:** Baseline cross-sectional correlations between components of well-being

	Experienced		Evaluative	Eudemonic	
	Positive affect	Negative affect	Life satisfaction	Control autonomy	Self-realisation
Positive affect					
Negative affect	−0.39***				
Life satisfaction	0.55***	−0.44***			
Control autonomy	0.51***	−0.38***	0.50***		
Self-realisation	0.67***	−0.44***	0.65***	0.57***	

*** P<0.001.

### Longitudinal associations

Results of the regression analyses are shown in table 3. Membership of education, arts or music groups, church or religious groups and sports or exercise groups at baseline were all associated with lower negative affect at follow-up, although the association with sports or exercise groups was attenuated when controlling for health-related covariates (table 3). Membership of church or religious groups was the only type of group membership associated with higher positive affect when controlling for demographic covariates, and this association held when considering health-related covariates too. Just education, arts or music groups were associated with higher life satisfaction, even when controlling for all covariates. Finally, sports or exercise groups were associated with higher control autonomy but (as with negative affect) the association was attenuated by health-related covariates. No groups were associated with higher self-realisation.

### Sensitivity analyses

These findings appeared to be robust to the alternative models tested in our sensitivity analyses. Controlling for how many times people took part in community groups did not appear to have much impact on results: associations between education, arts or music groups and church or other religious groups and higher positive and lower negative affect held and associations between education, arts or music groups and higher life satisfaction were only marginally attenuated (P=0.054) (see online supplementary table S1). The only difference related to sports and control autonomy: although the associations seen with model 1 were attenuated by model 2, once the number of groups people took part in was added into the model, the associations became significant again. Similarly, controlling for additional informal hobby and social engagement did not materially affect results: associations between education, arts or music groups and church or other religious groups and higher positive and lower negative affect held and associations between education, arts or music groups and higher life satisfaction were only marginally attenuated (P=0.065) (see online supplementary table S2). Excluding participants with mobility issues also did not affect the significance of responses (see online supplementary table S3), nor did weighting for non-response (see online supplementary table S4). When considering participation by age, those aged 65+ appeared to benefit the most from community group engagement, although there were still associations between both education, arts and music classes and higher life satisfaction, and church or religious groups and higher positive affect among those aged 55–64 (see online supplementary table S5).

### Cross-lagged analyses

Finally, our cross-lagged analyses, carried out for all significant results from our longitudinal analyses, explored whether well-being at baseline predicted group membership 10 years later in order to test for reverse causality. Results showed no significant association between baseline negative affect, positive affect or life satisfaction and either education, arts and music group or church or religious group membership 10 years later (data available on request).

**Table 3:** Regression coefficients showing associations between club and society membership and well-being

	Experienced						Evaluative			Eudemonic					
	Negative affect			Positive affect			Life satisfaction			Control autonomy			Self-realisation		
	OR	P value	CI	OR	P value	CI	β	P value	CI	β	P value	CI	β	P value	CI
Model 1															
Education, arts, music	**0.71**	**0.004**	**0.57** to **0.90**	0.98	0.87	0.77 to 1.25	**0.58**	**0.031**	**0.05** to **1.11**	0.20	0.12	–0.05 to 0.46	0.08	0.55	–0.18 to 0.34
Church or religious	**0.79**	**0.028**	**0.65** to **0.98**	**1.54**	**<0.001**	**1.25** to **1.90**	0.18	0.44	–0.28 to 0.65	0.06	0.62	–0.17 to 0.28	0.02	0.87	–0.21 to 0.25
Sports or exercise	**0.81**	**0.039**	**0.67** to **0.99**	1.15	0.17	0.94 to 1.41	0.16	0.49	–0.29 to 0.61	**0.25**	**0.023**	**0.04** to **0.47**	0.12	0.34	–0.11 to 0.33
Charitable	1.13	0.25	0.91 to 1.41	0.98	0.89	0.79 to 1.23	0.34	0.18	–0.15 to 0.82	–0.14	0.24	–0.38 to 0.10	0.05	0.69	–0.20 to 0.29
Social clubs	1.09	0.42	0.88 to 1.35	0.97	0.81	0.78 to 1.22	– 0.05	0.85	–0.53 to 0.44	–0.03	0.80	–0.27 to 0.20	0.04	0.74	–0.20 to 0.28
Political, union, environment	0.99	0.91	0.78 to 1.25	1.09	0.52	0.85 to 1.39	–0.17	0.55	–0.71 to 0.38	–0.10	0.46	–0.36 to 0.16	0.04	0.75	–0.23 to 0.31
Resident or neighbourhood	0.96	0.71	0.78 to 1.18	1.15	0.20	0.93 to 1.42	0.10	0.66	–0.36 to 0.57	–0.08	0.51	–0.15 to 0.30	0.11	0.34	–0.12 to 0.35
Other	0.90	0.29	0.75 to 1.09	0.97	0.79	0.80 to 1.19	–0.08	0.72	–0.51 to 0.35	–0.09	0.37	–0.11 to 0.30	0.10	0.35	–0.11 to 0.32
Model 2															
Education, arts, music	**0.73**	**0.007**	**0.57** to **0.92**	0.97	0.78	0.76 to 1.23	**0.55**	**0.041**	**0.02** to **1.08**	0.18	0.17	–0.07 to 0.43	0.07	0.62	–0.20 to 0.33
Church or religious	**0.79**	**0.03**	**0.65** to **0.98**	**1.54**	**<0.001**	**1.25** to **1.90**	0.18	0.45	–0.29 to 0.64	0.05	0.67	–0.18 to 0.27	0.02	0.85	–0.21 to 0.25
Sports or exercise	0.87	0.17	0.72 to 1.06	1.12	0.29	0.91 to 1.37	0.07	0.75	–0.37 to 0.52	0.18	0.11	–0.04 to 0.39	0.07	0.56	–0.15 to 0.29
Charitable	1.13	0.26	0.91 to 1.41	0.99	0.93	0.79 to 1.24	0.34	0.17	–0.15 to 0.83	–0.14	0.24	–0.38 to 0.09	0.06	0.65	–0.19 to 0.30
Social clubs	1.07	0.55	0.86 to 1.32	0.99	0.93	0.79 to 1.24	–0.01	0.95	–0.50 to 0.47	–0.01	0.95	–0.24 to 0.23	0.06	0.62	–0.18 to 0.30
Political, union, environment	0.99	0.96	0.78 to 1.26	1.08	0.56	0.84 to 1.38	–0.18	0.51	–0.73 to 0.36	–0.11	0.41	–0.37 to 0.15	0.03	0.82	–0.24 to 0.30
Resident or neighbourhood	0.95	0.64	0.77 to 1.17	1.15	0.21	0.93 to 1.42	0.11	0.64	–0.35 to 0.58	0.08	0.49	–0.15 to 0.30	0.12	0.32	–0.11 to 0.34
Other	0.91	0.35	0.76 to 1.10	0.97	0.75	0.79 to 1.18	–0.10	0.64	–0.53 to 0.33	0.08	0.45	–0.13 to 0.29	0.10	0.37	–0.11 to 0.31

Bold signifies a P value of 0.05 or lower (precise P values are shown in the table).

Model 1 adjusted for baseline well-being, sex, age, marital status, ethnicity, educational attainment, employment status and wealth. Model 2 additionally adjusted for eyesight, hearing, having a chronic health condition (including cancer, chronic obstructive pulmonary disease, arthritis, diabetes or angina) or having had a chronic health condition in the last two years (including cancer and a stroke), and chronic pain.

OR, OR (logistic regression models); β, beta coefficient (linear regression models).

## Discussion

This study aimed to identify whether membership of different types of community groups (such as clubs and societies) was longitudinally associated with well-being 10 years later in adults aged 55 and older. Two types of community groups had significant associations when controlling for covariates: education, arts and music classes (associated with both lower negative affect and higher life satisfaction) and church or religious groups (associated with both lower negative affect and higher positive affect). Sports groups, gym and exercise classes were initially associated with lower negative affect and higher control autonomy, but these associations appeared to be linked with health-related covariates and more frequent attendance was required for effects to be seen.

Consequently, just two types of group membership were found to have robust associations with well-being. In relation to church or religious groups, our results replicated findings from previous studies in showing associations with negative affect.^14^ However, unlike other studies, we did not find associations with life satisfaction.^25^ Previous studies have suggested that religious groups have more robust effects on well-being than secular groups.^26^ However, our results in fact showed that education, arts and music groups were also associated with well-being with comparable effect sizes. So it would not appear that religion is a required component for group engagement to have beneficial effects. In relation to ‘education, arts or music groups or evening classes’, our findings extend some previous work that has been done using the ELSA dataset looking at educational activities as predictors of well-being.^27–29^ These previous analyses have included the same variable on education, arts or music groups but over a shorter follow-up period. Broadly, they have found some similar results, but by extending the follow-up from 5 years to 10 years, we found that associations with eudemonic well-being found at 5-year follow-up have disappeared at 10-year follow-up. As such, it appears that education, arts or music groups are only associated with hedonic, not eudemonic, well-being over longer periods.

An important consideration with both church/religious groups and education/arts/music groups is whether the effects found were the result of group engagement and associated social mechanisms or longer-term factors such as personality or processes such as religiosity and lifelong learning. While it was not possible to test this specifically within these analyses, it is of note that interventional studies involving engagement in community arts and music groups for those who were not previously engaged have found significant changes in different dimensions of well-being.^18 30^ This suggests that membership of arts/music groups can causally modify well-being and effects are not limited to those who have had lifelong involvement.

Despite the apparent benefit of engaging in education, arts and music groups and church or religious groups, fewer than a quarter or older adults do either of these activities and there is such a clear social gradient in participation, despite the fact such community groups can be free or very low cost to join and are widely available. It has been proposed that policies are put in place to support family, work, education and religious communities given that they have been so strongly linked with human flourishing.^31^ While engagement with religious groups is no doubt determined largely by one’s own religious beliefs so it is harder to intervene on, the data presented here suggest that engagement with education, arts or music classes should be supported too with the same aim. Interventions or policies to encourage engagement could be designed to try and directly support well-being in older adults who may not normally access these community group resources and to extend the observational research presented here. Pilot models offering some of these activities ‘on prescription’ through primary care to hard-to-reach older adults in the UK have shown promising results.^32^

This study has a number of strengths, including a large, nationally representative sample of older adults, a direct simultaneous comparison of eight different types of group membership, a consideration of associations with five different types of well-being and a 10-year longitudinal follow-up period. But as the data are observational, causality cannot be assumed. Nevertheless, analyses did account for all identified confounders and cross-lagged analyses showed a predictive relationship of group membership and well-being, but not vice versa, suggesting that reverse causality was not the driver of the effects noted here. Additionally, we were restricted by the existing variables on community group membership, which, as discussed above, comprised some broad categories. So future studies are encouraged, in particular looking in more detail at more specific types of arts, education, music and religious groups and involving interventional designs to identify whether referring older adults to such community groups could causally lead to enhanced well-being. This analysis also focused on an exposure and follow-up. Future studies could consider how patterns of engagement and, in particular, sustained engagement are associated with well-being. Finally, this analysis looked at ‘membership’ without specifying a frequency of engagement/attendance for each specific group. Although our sensitivity analyses showed that more frequent engagement with community groups overall did not lead to an attenuation of results, it remains to be identified what frequency of engagement/attendance with specific groups such as education, arts or music groups or church or other religious groups is required for associations to be found.

Overall, this study suggests that education, arts or music classes and church or religious groups may support well-being in older age. Given that those activities are seemingly less engaged with by those lower down the socioeconomic scale and with lower educational attainment, it will be key to examine further barriers to engagement so that these community activities can be equally drawn on to support health and well-being by all.

**What is already known on this subject**

- Improving well-being in older age is recognised as a public health priority, not least because poor well-being is associated with a range of physical health conditions.
- Some previous studies have suggested that membership of community groups can enhance well-being, but it remains unclear which types of groups are associated with which aspects of well-being.

**What this study adds**

- This study demonstrates the prevalence of engagement in community groups among older adults in England, highlighting the influence of demographic and socioeconomic factors on attendance.
- This study also shows that specifically attending education, arts or music classes is longitudinally associated with higher experienced and evaluative well-being between baseline and follow-up 10 years later.
- It shows that attending church or religious groups is longitudinally associated with higher experienced well-being.
- These findings suggest that joining community groups could be more actively promoted among older adults to support well-being.

## References

[1] WHO. World Report on Ageing and Health. World Health Organization 2015.

[2] Martín-María N, Miret M, Caballero FF, et al The Impact of Subjective Well-being on Mortality: A Meta-Analysis of Longitudinal Studies in the General Population. Psychosom Med 2017;79:565–75. 10.1097/PSY.0000000000000444 28033196

[3] Steptoe A, Deaton A, Stone AA Subjective wellbeing, health, and ageing. The Lancet 2015;385:640–8. 10.1016/S0140-6736(13)61489-0 PMC4339610 25468152

[4] Stone A, Mackie C, Subjective Well-Being: Measuring Happiness, Suffering, and Other Dimensions of Experience. Washington, DC: National Academies Press (US), 2013. 24432436

[5] Hyde M, Wiggins RD, Higgs P, et al A measure of quality of life in early old age: the theory, development and properties of a needs satisfaction model (CASP-19). Aging Ment Health 2003;7:186–94. 10.1080/1360786031000101157 12775399

[6] Jivraj S, Nazroo J, Vanhoutte B, et al Aging and Subjective Well-Being in Later Life. J Gerontol B Psychol Sci Soc Sci 2014;69:930–41. 10.1093/geronb/gbu006 24569002 PMC4296137

[7] Holt-Lunstad J, Smith TB, Layton JB, et al and Mortality Risk: A Meta-analytic Review. PLOS Med 2010;7:e1000316. 20668659 10.1371/journal.pmed.1000316 PMC2910600

[8] Haslam SA, Jetten J, Postmes T, et al Social Identity, Health and Well-Being: An Emerging Agenda for Applied Psychology. Appl Psychol 2009;58:1–23. 10.1111/j.1464-0597.2008.00379.x

[9] Halleröd B, Örestig J, Stattin M Leaving the labour market: the impact of exit routes from employment to retirement on health and wellbeing in old age. Eur J Ageing 2013;10:25–35. 10.1007/s10433-012-0250-8 28804280 PMC5549229

[10] Iyer A, Jetten J, Tsivrikos D, et al The more (and the more compatible) the merrier: multiple group memberships and identity compatibility as predictors of adjustment after life transitions. Br J Soc Psychol 2009;48:707–33. 10.1348/014466608X397628 19200408

[11] Jetten J, O’Brien A, Trindall N Changing identity: predicting adjustment to organizational restructure as a function of subgroup and superordinate identification. Br J Soc Psychol 2002;41:281–97. 10.1348/014466602760060147 12133229

[12] DE VAUS D, Wells Y, KENDIG HAL, et al Does gradual retirement have better outcomes than abrupt retirement? Results from an Australian panel study. Ageing Soc 2007;27:667–82. 10.1017/S0144686X07006228

[13] MacKean R, Abbott-Chapman J Older people’s perceived health and wellbeing: The contribution of peer-run community-based organisations. Health Sociol Rev 2012;21:47–57. 10.5172/hesr.2012.21.1.47

[14] Kaushal A, Cadar D, Stafford M, et al PP66 Lifetime influences of religious attendance and beliefs on mental health and wellbeing in older age. J Epidemiol Community Health 2015;69:A81.1–A81. 10.1136/jech-2015-206256.163

[15] Jenkinson CE, Dickens AP, Jones K, et al Is volunteering a public health intervention? A systematic review and meta-analysis of the health and survival of volunteers. BMC Public Health 2013;13:773 10.1186/1471-2458-13-773 23968220 PMC3766013

[16] Binder M, Blankenberg A-K Environmental concerns, volunteering and subjective well-being: Antecedents and outcomes of environmental activism in Germany. Ecol Econ 2016;124:1–16. 10.1016/j.ecolecon.2016.01.009

[17] Milligan C, Neary D, Payne S, et al Older men and social activity: a scoping review of Men’s Sheds and other gendered interventions. Ageing Soc 2016;36:895–923. 10.1017/S0144686X14001524

[18] Coulton S, Clift S, Skingley A, et al Effectiveness and cost-effectiveness of community singing on mental health-related quality of life of older people: randomised controlled trial. Br J Psychiatry 2015;207:250–5. 10.1192/bjp.bp.113.129908 26089304

[19] Eime RM, Harvey JT, Brown WJ, et al Does sports club participation contribute to health-related quality of life? Med Sci Sports Exerc 2010;42:1022–8. 10.1249/MSS.0b013e3181c3adaa 19996991

[20] Thoits PA Multiple identities and psychological well-being: a reformulation and test of the social isolation hypothesis. Am Sociol Rev 1983;48:174–87. 10.2307/2095103 6859677

[21] Vanhoutte B The Multidimensional Structure of Subjective Well-Being In Later Life. J Popul Ageing 2014;7:1–20. 10.1007/s12062-014-9092-9 25089162 PMC4102758

[22] Radloff LS The CES-D Scale: A Self-Report Depression Scale for Research in the General Population. Appl Psychol Meas 1977;1:385–401.

[23] Vanhoutte B Measuring subjective well-being: a review. Manchester: CCSR, University of Manchester, 2012 https://www.escholar.manchester.ac.uk/uk-ac-man-scw:195190

[24] Diener E, Emmons RA, Larsen RJ, et al The Satisfaction With Life Scale. J Pers Assess 1985;49:71–5. 10.1207/s15327752jpa4901_13 16367493

[25] Warr P, Butcher V, Robertson I Activity and psychological well-being in older people. Aging Ment Health 2004;8:172–83. 10.1080/13607860410001649662 14982722

[26] Acevedo GA, Ellison CG, Xu X Is It Really Religion? Comparing the Main and Stress-buffering Effects of Religious and Secular Civic Engagement on Psychological Distress. Soc Ment Health 2014;4:111–28. 10.1177/2156869313520558

[27] Jenkins A Participation in Learning and Depressive Symptoms. Educ Gerontol 2012;38:595–603. 10.1080/03601277.2011.595325

[28] Jenkins A Participation in learning and wellbeing among older adults. Int J Lifelong Educ 2011;30:403–20. 10.1080/02601370.2011.570876

[29] Jenkins A, Mostafa T The effects of learning on wellbeing for older adults in England. Ageing Soc 2015;35:2053–70. 10.1017/S0144686X14000762

[30] Osgood NJ, Meyers BS, Orchowsky S The Impact of Creative Dance and Movement Training on the Life Satisfaction of Older Adults: An Exploratory Study. J Appl Gerontol 1990;9:255–65. 10.1177/073346489000900302

[31] VanderWeele TJ On the promotion of human flourishing. Proc Natl Acad Sci U S A 2017;114:8148–56. 10.1073/pnas.1702996114 28705870 PMC5547610

[32] Crone DM, O’Connell EE, Tyson PJ, et al ’Art Lift' intervention to improve mental well-being: an observational study from U.K. general practice. Int J Ment Health Nurs 2013;22:279–86. 10.1111/j.1447-0349.2012.00862.x 22897659

