# Supplementary material for: Community group membership and multidimensional subjective well-being in older age
Source: J Epidemiol Community Health. 2018 Feb 9;72(5):376–82. doi: 10.1136/jech-2017-210260 (PMC5909739; doi:10.1136/jech-2017-210260)
Supplement: Supplementary file 1 [file jech-2017-210260supp001.pdf]

Table S1: Regression coefficients showing associations between club and society membership and wellbeing, additionally controlling for frequency of club attendance

	Experienced				Evaluative		Eudemonic			
	Negative affect		Positive affect		Life satisfaction		Control-autonomy		Self-realisation	
	OR	CI	OR	CI	β	CI	β	CI	β	CI
Education, arts, music	**0.73**	**0.58-0.92**	0.94	0.74-1.20	0.52	-0.01-1.05	0.17	-0.09-0.42	0.05	-0.21-0.32
Church or religious	**0.81**	**0.65-1.00**	**1.44**	**1.16-1.79**	0.10	-0.37-0.58	0.02	-0.21-0.25	-0.01	-0.25-0.22
Sports or exercise	0.88	0.72-1.07	1.08	0.88-1.33	0.04	-0.41-0.49	**0.17**	**0.05-0.38**	0.05	-0.17-0.28
Charitable	1.16	0.92-1.45	0.89	0.71-1.13	0.23	-0.28-0.74	-0.18	-0.42-0.06	0.01	-0.25-0.26
Social clubs	1.07	0.87-1.33	0.97	0.77-1.21	-0.04	-0.52-0.44	-0.02	-0.25-0.22	0.05	-0.19-0.29
Political, union, environment	1.00	0.78-1.26	1.07	0.83-1.37	-0.20	-0.74-0.35	-0.11	-0.38-0.15	0.03	-0.24-0.30
Resident or neighbourhood	0.96	0.78-1.18	1.10	0.89-1.37	0.07	-0.40-0.53	-0.06	-0.16-0.29	0.10	-0.14-0.33
Other	0.93	0.76-1.12	0.90	0.74-1.10	-0.22	-0.62-0.26	-0.05	-0.16-0.26	0.06	-0.16-0.28

OR=odds ratio (logistic regression models); β=beta coefficient (linear regression models); CI=confidence intervals. Adjusted for baseline wellbeing, sex, age, marital status, ethnicity, educational attainment, employment status, wealth, eyesight, hearing, having a chronic health condition (including cancer, COPD, arthritis, diabetes or angina) or having had a chronic health condition in the last 2 years (including cancer and a stroke), chronic pain and frequency of club attendance.

Table S2: Regression coefficients showing associations between club and society membership and wellbeing, additionally controlling for informal engagement

	Experienced				Evaluative		Eudemonic			
	Negative affect		Positive affect		Life satisfaction		Control-autonomy		Self-realisation	
	OR	CI	OR	CI	β	CI	β	CI	β	CI
Education, arts, music	**0.74**	**0.59-0.94**	0.98	0.76-1.25	0.51	-0.02-1.04	0.16	-0.10-0.41	0.03	-0.23-0.30
Church or religious	**0.80**	**0.65-0.98**	**1.55**	**1.25-1.91**	0.17	-0.30-0.63	0.04	-0.18-0.26	0.01	-0.22-0.24
Sports or exercise	0.89	0.73-1.08	1.12	0.91-1.37	0.02	-0.43-0.48	0.15	-0.07-0.37	0.02	-0.20-0.25
Charitable	1.13	0.91-1.41	0.98	0.78-1.23	0.32	-0.17-0.81	-0.15	-0.38-0.09	0.04	-0.20-0.29
Social clubs	1.07	0.86-1.32	0.98	0.78-1.23	-0.03	-0.51-0.46	-0.01	-0.25-0.22	0.05	-0.19-0.29
Political, union, environment	1.00	0.79-1.27	1.08	0.84-1.39	-0.20	-0.74-0.34	-0.12	-0.38-0.14	0.02	-0.25-0.29
Resident or neighbourhood	0.95	0.78-1.17	1.14	0.92-1.42	-.10	-0.37-0.56	0.07	-0.15-0.30	0.11	-0.13-0.34
Other	0.93	0.77-1.12	0.97	0.80-1.18	-0.13	-0.57-0.29	0.06	-0.15-0.27	0.06	-0.15-0.28

OR=odds ratio (logistic regression models); β=beta coefficient (linear regression models); CI=confidence intervals. Adjusted for baseline wellbeing, sex, age, marital status, ethnicity, educational attainment, employment status, wealth, eyesight, hearing, having a chronic health condition (including cancer, COPD, arthritis, diabetes or angina) or having had a chronic health condition in the last 2 years (including cancer and a stroke), chronic pain, informal hobby/pastime engagement and informal social engagement with relatives.

Table S3: Regression coefficients showing associations between club and society membership and wellbeing, excluding participants with mobility issues affecting their walking

	Experienced				Evaluative		Eudemonic			
	Negative affect		Positive affect		Life satisfaction		Control-autonomy		Self-realisation	
	OR	CI	OR	CI	β	CI	β	CI	β	CI
Education, arts, music	**0.71**	**0.56-0.90**	0.95	0.74-1.21	**0.59**	**0.06-1.13**	0.18	-0.08-0.43	0.08	-0.18-0.35
Church or religious	**0.83**	**0.67-1.02**	**1.49**	**1.20-1.86**	0.11	-0.36-0.59	0.02	-0.21-0.25	0.03	-0.21-0.27
Sports or exercise	0.86	0.71-1.06	1.11	0.91-1.37	0.11	-0.34-0.56	0.18	-0.04-0.39	0.08	-0.14-0.31
Charitable	1.12	0.89-1.39	1.02	0.81-1.28	0.34	-0.16-0.84	-0.12	-0.36-0.12	0.08	-0.17-0.33
Social clubs	1.05	0.84-1.31	0.97	0.77-1.22	-0.12	-0.62-0.37	-0.02	-0.22-0.25	0.01	-0.23-0.26
Political, union, environment	1.00	0.78-1.27	1.09	0.85-1.40	-0.14	-0.69-0.41	-0.10	-0.37-0.16	0.03	-0.24-0.30
Resident or neighbourhood	0.97	0.78-1.20	1.15	0.92-1.43	0.16	-0.31-0.63	0.07	-0.16-0.30	0.20	-0.03-0.44
Other	0.93	0.77-1.13	0.99	0.81-1.21	-0.15	-0.58-0.29	0.07	-0.14-0.28	0.08	-0.13-0.30

OR=odds ratio (logistic regression models); β=beta coefficient (linear regression models); CI=confidence intervals. Adjusted for baseline wellbeing, sex, age, marital status, ethnicity, educational attainment, employment status, wealth, eyesight, hearing, having a chronic health condition (including cancer, COPD, arthritis, diabetes or angina) or having had a chronic health condition in the last 2 years (including cancer and a stroke) and chronic pain. N=2,385.

Table S4: Regression coefficients showing associations between club and society membership and wellbeing, weighted to account for non-response

	Experienced				Evaluative		Eudemonic			
	Negative affect		Positive affect		Life satisfaction		Control-autonomy		Self-realisation	
	OR	CI	OR	CI	β	CI	β	CI	β	CI
Education, arts, music	**0.71**	**0.56-0.90**	0.95	0.75-1.22	**0.56**	**0.07-1.06**	0.17	-0.07-0.41	0.05	-0.21-0.31
Church or religious	**0.77**	**0.62-0.95**	**1.50**	**1.21-1.87**	0.12	-0.37-0.61	0.02	-0.21-0.25	0.01	-0.23-0.25
Sports or exercise	0.89	0.73-1.09	1.12	0.91-1.37	0.06	-0.38-0.51	0.20	-0.02-0.41	0.06	-0.17-0.28
Charitable	1.18	0.95-1.47	0.99	0.79-1.25	0.37	-0.12-0.85	-0.18	-0.42-0.06	0.02	-0.23-0.28
Social clubs	1.04	0.83-1.29	0.99	0.78-1.24	-0.01	-0.50-0.48	-0.02	-0.27-0.22	0.06	-0.18-0.30
Political, union, environment	1.00	0.78-1.28	1.05	0.82-1.35	-0.14	-0.68-0.41	-0.13	-0.39-0.14	0.02	-0.23-0.28
Resident or neighbourhood	0.96	0.78-1.19	1.13	0.91-1.40	0.11	-0.36-0.58	0.07	-0.16-0.29	0.12	-0.12-0.36
Other	0.92	0.76-1.12	0.96	0.79-1.17	-0.14	-0.57-0.28	0.09	-0.12-0.30	0.09	-0.13-0.30

OR=odds ratio (logistic regression models); β=beta coefficient (linear regression models); CI=confidence intervals. Adjusted for baseline wellbeing, sex, age, marital status, ethnicity, educational attainment, employment status, wealth, eyesight, hearing, having a chronic health condition (including cancer, COPD, arthritis, diabetes or angina) or having had a chronic health condition in the last 2 years (including cancer and a stroke) and chronic pain. N=2,385.

Table S5: Regression coefficients showing associations between club and society membership and wellbeing, split by age

	Experienced				Evaluative		Eudemonic			
	Negative affect		Positive affect		Life satisfaction		Control-autonomy		Self-realisation	
	OR	CI	OR	CI	β	CI	β	CI	β	CI
**Aged 55-64**										
Education, arts, music	0.82	0.59-1.12	1.08	0.77-1.50	**0.75**	**0.04-1.46**	0.16	-0.17-0.48	0.16	-0.18-0.50
Church or religious	0.83	0.62-1.11	**1.38**	**1.03-1.85**	-0.15	-0.79-0.49	-0.04	-0.33-0.25	-0.23	-0.54-0.07
Sports or exercise	0.92	0.72-1.19	1.06	0.81-1.38	0.15	-0.41-0.72	0.23	-0.03-0.49	0.06	-0.21-0.34
Charitable	1.16	0.87-1.55	1.08	0.80-1.45	0.71	-0.07-1.35	-0.17	-0.47-0.12	0.31	0.003-0.62
Social clubs	1.09	0.82-1.46	1.14	0.84-1.55	0.11	-0.52-0.75	-0.21	-0.08-0.51	0.27	-0.04-0.58
Political, union, environment	0.80	0.59-1.08	1.25	0.91-1.72	-0.24	-0.91-0.43	-0.22	-0.53-0.09	0.05	-0.28-0.37
Resident or neighbourhood	0.97	0.73-1.28	1.4	0.78-1.39	0.37	-0.25-0.99	0.22	-0.06-0.50	0.21	-0.09-0.51
Other	0.98	0.76-1.26	0.80	0.61-1.04	-0.26	-0.82-0.30	-0.05	-0.30-0.21	-0.09	-0.36-0.18
**Aged 65+**										
Education, arts, music	**0.64**	**0.45-0.90**	0.87	0.60-1.25	**0.43**	**-0.38-1.23**	0.22	-0.19-0.63	-0.01	-0.43-0.41
Church or religious	**0.78**	**0.58-1.06**	**1.80**	**1.32-2.45**	0.67	-0.02-1.36	0.19	-0.16-0.54	0.36	-0.004-0.71
Sports or exercise	0.80	0.58-1.09	1.18	0.85-1.64	0.05	-0.68-0.79	0.14	-0.24-0.51	0.09	-0.29-0.47
Charitable	1.05	0.75-1.48	0.90	0.63-1.27	-0.22	-0.99-0.54	-0.16	-0.55-0.23	-0.30	-0.69-0.10
Social clubs	0.98	0.70-1.37	0.84	0.59-1.18	-0.20	-0.95-0.54	-0.29	-0.67-0.09	-0.19	-0.58-0.19
Political, union, environment	1.37	0.91-2.07	0.90	0.59-1.38	0.17	-0.76-1.10	0.14	-0.34-0.61	0.11	-0.37-0.59
Resident or neighbourhood	0.91	0.67-1.25	1.28	0.95-1.77	-0.31	-1.03-0.41	-0.12	-0.49-0.26	-0.01	-0.38-0.36
Other	0.84	0.63-1.12	1.23	0.91-1.66	-0.04	-0.71-0.63	0.20	-0.14-0.54	0.31	-0.04-0.65

OR=odds ratio (logistic regression models); β=beta coefficient (linear regression models); CI=confidence intervals. Adjusted for baseline wellbeing, sex, age, marital status, ethnicity, educational attainment, employment status, wealth, eyesight, hearing, having a chronic health condition (including cancer, COPD, arthritis, diabetes or angina) or having had a chronic health condition in the last 2 years (including cancer and a stroke) and chronic pain. Aged 55-64, n=1,504. Aged 65+, n=1,044.
